# Non-Union Treatment in the Shoulder, Arm, Wrist, and Fingers: A Multicentre Retrospective Study Comparing Conventional Treatment with the Human Allogeneic Cortical Bone Screw (Shark Screw^®^)

**DOI:** 10.3390/life15091421

**Published:** 2025-09-10

**Authors:** Elisabeth Huber, Gerd Jakob, Wolfgang Palle, Gudrun H. Borchert, Klaus Pastl

**Affiliations:** 1 DOKH Friesach, St Veit Str. 12, A-9360 Friesach, Austria; elisabeth.huber@dokh.at (E.H.); wolfgang.palle@aon.at (W.P.); 2 Landeskrankenhaus Villach, Nikolaigasse 43, A-9500 Villach, Austria; gerd.jakob@me.com; 3 Dr. Borchert Medical Information Management, Egelsbacher Str. 39e, D-63225 Langen, Germany; gudrun.borchert@borchert-medical.com; 4 Klinik Diakonissen Linz, Weißenwolffstraße 13, A-4020 Linz, Austria

**Keywords:** human allogeneic cortical bone screw (Shark Screw^®^), metal hardware removal, non-union, pseudarthrosis, upper extremity

## Abstract

Successful non-union therapy consists of a combination of optimizing mechanical stability and activating biological factors. The conventional method for treating non-union is debridement and stabilization with metal hardware. The human allogeneic cortical bone screw (Shark Screw^®^) merges human cortical bone properties with screw stability, addressing non-union surgery principles by integrating mechanical and biological aspects. The objective of this retrospective study was to compare the clinical and radiological outcomes of the conventional method with those of the new method using the Shark Screw^®^. This retrospective, multicentre, level III study included 41 patients with non-unions in upper extremities, 11 treated with the conventional method (metal hardware ± graft), and 30 patients with the Shark Screw^®^ (±graft). Patient demographics, non-union location, autograft and/or allograft use, follow-up time, complications, union rate, time to union, and time to return to work were recorded. Follow-up was 18 months in the conventional group and 10 months in the Shark Screw^®^ group. The union rate was 72.7% in the conventional group and 96.7% in the Shark Screw^®^ group. Time to union was significantly shorter in the Shark Screw^®^ group. In the conventional group, the complication rate was 36%, and it was 3.4% in the Shark Screw^®^ group. Hardware removal in the conventional group was 64%, and it was 0% in the Shark Screw^®^ group. The Shark Screw^®^ presents a reliable option for treating non-unions in the shoulder, forearm, hand, and fingers.

## 1. Introduction

Physiological fracture healing is a biologically outstanding achievement that can be disturbed by many factors [1]. The cause of non-union seems to be the interaction of various biological and biomechanical factors with systemic and local interactions [1,2]. Regarding local causes, unsuitable or faulty stabilization, possibly with long periods of reduced loading, pronounced soft-tissue damage, reduced blood supply in the affected region and traumatic or iatrogenic destruction of the periosteum, former radiation, and infection are believed to play a role in the fracture failing to heal [1,2]. A recent study by Patterson et al. [3] demonstrates that age at surgery, dominant hand injury, and previous surgery on the affected scaphoid are risk factors for scaphoid non-union. Gagnon et al. [4] summarize the difficulty encountered in treating non-unions and obtaining a level I study due to issues involving bone defects, biology, mechanical stability, surgical technique, and host factors [4] because of the different procedures necessary to overcome non-union. Non-union results in prolonged pain and reduced functionality [5]. Socioeconomic costs are incurred by the complex treatment strategies involving operations [5,6,7,8]. A combination of optimizing mechanical stability and activating biological factors is most important to overcome non-union [1,8,9]. The application of autologous cancellous bone is often used to biologically induce fracture healing [2,10,11,12], but its limited availability and the high donor-site morbidity [10,12] represent a great disadvantage. The site of a non-union can be augmented with allogeneic bone graft. Allografts are an alternative and act as autografts [10,13]. The conventional method for treating non-union is the debridement of the non-union and stabilization with metal plates and screws. However, metal hardware leads to complications and a potential second operation for hardware removal. Complication rates between 30 and 60% are reported after total wrist arthrodesis, and the re-operation rate is between 19 and 64% [14]. Nwosu et al. note in their recent review that 3–31% [14] of these constitute hardware-related complications. The hardware removal rate was recorded to be between 6.8% after fixation of the volar locking plate for distal radius fractures in adults [14], and up to 77% after arthrodesis for primary osteoarthritis of the trapezoid-metacarpal joint [15,16,17,18]. Avoiding hardware removal and donor-site morbidity while obtaining comparable results to conventional methods would decrease economic burden and entail a lower risk for the patient.

The non-union rate in the shoulder is reported to be 3.4% [19]. Non-union in long bones is recorded to be up to 30% [1]. Vanderkarr et al. [20] describe a non-union rate of 7.2% for the humerus. In the forearm, non-unions are reported to occur between 0 and 60% of the time [11,21,22,23]. Non-union rates are described to be 4% for the radius, with rates of 7% for the ulna [2] and 11% for the hand [9]. For the scaphoid, the incidence of non-unions is reported to be between 0% and 18%, and up to 63% in some cases [12,21,22,23,24]. Treating scaphoid non-unions arthroscopically with autografts from the distal radius and four pins resulted in 93.5% union [25]. Dislocated fractures, in particular, seem to be prone to non-union [7,26,27]. Displaced scaphoid fractures are at risk for non-union due to a variety of factors, including interfragmentary instability, retrograde vascular supply, and a lack of soft-tissue attachments on a largely cartilaginous surface [27,28]. Non-union rate after arthrodesis of the proximal interphalangeal joint of the finger is reported to be 3.9% using the compression screw and 8.6% after interosseous wiring [29]. Fractures with severe soft-tissue trauma are also at increased risk of non-union [20].

To avoid progression to arthrosis of the scaphoid, there are three major aims, namely restoration of the form of the scaphoid, restoration of the carpal alignment, and bony consolidation of the scaphoid [21]. Despite improvements in surgical procedures treating non-union, there is no consensus over the optimal treatment option.

For certain age groups, Mills at al. [30] described a non-union rate up to 9%, with 80 non-unions per 1000 fractures in the shoulder per year for the age group between 35 and 45 [30]. This is mainly true for male patients; for female patients, an increase in incidence is reported with age [9,31].

The human allogeneic cortical bone screw (Shark Screw^®^) represents a new option in treating non-unions [8]. The human cortical bone screw provides close contact between allograft and host bone, which is required for revascularisation and healing, as described by Basile et al. [32] and confirmed by Brcic for the human allogeneic cortical bone screw [33]. It has been reported that (metal) screw fixation disrupts the internal vascularity [34,35] more than a K-wire and hence reduces the potential benefits of vascular grafts [34]. Perren et al. [35] notes that a plate used for radius fixation, for example, inhibits blood reaching or leaving the bone. This complication is avoided by using the cortical bone screw because vascularization is not disrupted. The success of the graft may also depend on the quality of the bone bed from which most of the revascularization arises [33,36]. The processing, preservation, and sterilization (via irradiation or chemical sterilization) of allograft bone may influence its biophysical and general biological properties [36]. The goal when using bone allograft is to initiate a healing response from the host bed that will produce new bone at the host–graft interface and within the porous body of the graft material [36]. The mechanical stability of the graft is of vital importance [36], and this stability is provided by the Shark Screw^®^, designed as a set screw. For the human allogeneic cortical bone screw, this process of ensuring mechanical stability was illustrated after 10 weeks [33]. Recent publications show that, when using the Shark Screw^®^, the union rate is between 94 and 100% for the treatment of fractures, arthrodesis, and non-union [37,38,39,40], and thus similar to union rates with conventional methods.

The multicentred retrospective data analysis presented here aims to show the diversity of cases where the Shark Screw^®^ can be used and to compare the outcome with that of the conventional method in upper extremities. We recorded patient demographics, follow-up time, union rate, time to union, time to return to work, complications, and percentage of hardware removal. We collected data from the clavicula, humerus, radius, ulna, the scaphoid, and the finger joints. Our hypothesis is that the human allogeneic cortical bone screw is as effective as the conventional method for the treatment of non-unions (pseudarthrosis).

## 2. Materials and Methods

Approval from the local institutional review boards (IRBs) was received before the study’s commencement, and the reference numbers are as follows: 1146/2023 (Ethik-Kommission of the Johannes Keppler University Linz), and M2023-25 (Ethik-Kommission Kärnten). The study was conducted in accordance with the Declaration of Helsinki. Informed consent to participate was waived by the institutional review boards. Informed consent is not mandatory for retrospective studies. The raw data are summarized in Supplementary Materials—Table S1.

### 2.1. Study Design and Patient Population

**Figure 1:**
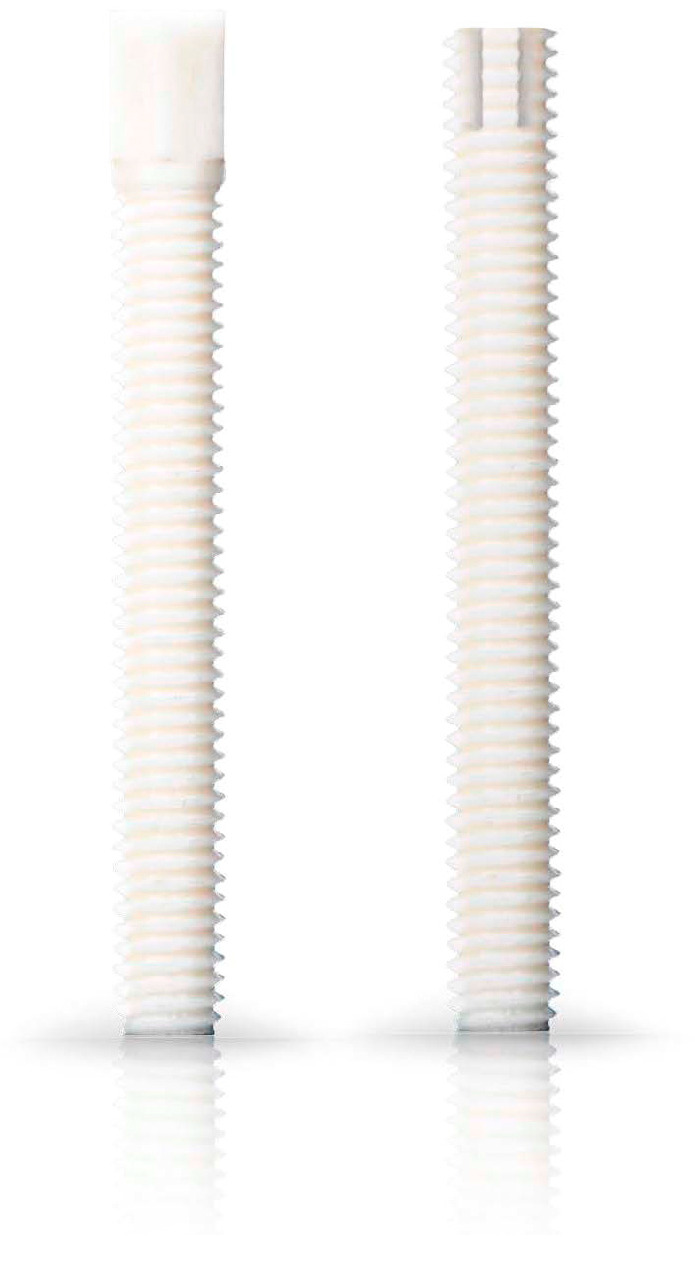
Examples of Shark Screws^®^: Shark Screw^®^ CUT and Shark Screw^®^ DIVER, used for the surgery.

This study was designed as a retrospective multicentred study. Clinical and radiographic data were collected from the medical charts of 41 patients who underwent non-union surgery on the shoulder, arm, and fingers between 2016 and 2022. The specific treatment method for non-union was not a selection criterion. Our study included a total of 41 patients, with 11 patients (27%) in the conventional treatment group and 30 patients (73%) in the Shark Screw^®^ group. Patient demographics are presented in Table 1. Patients from two treatment groups were evaluated: the conventional treatment involving metal hardware (screws/plates) (different providers) with or without bone graft, and the Shark Screw^®^ procedure (Table 2). Figure 1 shows the Shark Screw^®^ (Surgebright GmbH, Lichtenberg, Austria). The comorbidities included diabetes mellitus type II, COPD, mutilating chronic polyarthritis, arterial hypertension, adenocarcinoma, (allergic) bronchial asthma, lupus erythematosus, osteoporosis, foramen ovale, deep vein thrombosis, varicose veins, and benign prostatic hyperplasia.

### 2.2. Inclusion Criteria

To be eligible for inclusion in the study, patients were required to have at least one follow-up visit at least six months post-surgery or until bone healing was documented. These cases encompassed non-union after elective procedures (arthrodesis and osteotomy), after surgical fracture treatment, and after conservative fracture treatment (Table 1).

### 2.3. Exclusion Criteria

We excluded non-unions related to tumour-related cases.

### 2.4. Surgical Procedures

Surgical procedures vary with the location of the non-union. When using the cortical bone screw, previous surgical procedures have been described for the hand [37], for scaphoid fractures [39], and for distal interphalangeal joint arthrodesis [40]. We recorded the following parameters: non-union location for revision (Table 2), autograft and allograft use, follow-up time, complications, union rate, time to union, and time to return to work (Table 3).

### 2.5. Complications

We defined non-union, partial union after 6 months, osteonecrosis, screw loosening, or screw breakage as a complication.

### 2.6. Statistics

Data are presented as mean ± SD. Due to the non-Gaussian distribution of the data, a non-parametric Kruskal–Wallis ANOVA was used for calculating significant differences in quantitative values. Ordinal values were evaluated with contingency tables using Fisher’s exact test for significance. Sample size calculation was performed for time to union and for return to work. A power of 0.8 was determined sufficient. For time to union, sample sizes of 6 and 12 were calculated for the conventional and Shark Screw^®^ groups, respectively. For return to work, 11 and 21 were the sample sizes required to reach a power of 0.8. for the conventional and Shark Screw^®^ groups, respectively. For the calculation, the programme G-Power was used. A *p*-value < 0.05 was considered to be significant, with a power > 0.8. All remaining statistical analyses were performed using Origin Pro statistical software (OriginPro, version 2025; OriginLab Corporation, Northampton, MA, USA).

## 3. Results

### 3.1. Patient Demographics and Clinical Characteristics

The patients’ average age was 41 years in the conventional group and 56 years in the Shark Screw^®^ group; this difference was statistically significant (*p* = 0.00669). Patients in the Shark Screw^®^ group were significantly older. The patients’ average BMI was 25 kg/m^2^ in both groups. Smokers made up 45% of the conventional group and 25% of the Shark Screw^®^ group, and 54% and 47% were male patients in the conventional and Shark Screw^®^ groups, respectively (Table 1).

The anatomic locations of non-union for revision are summarized in Table 2.

For the conventional group, metal screws were used in four cases, plates in three cases, K-wires in two cases, a mini-fixateur in one, and in one case, a prothesis was utilized. In the Shark Screw^®^ group, the metal hardware was left in in one case. Autograft was used only in six cases in the conventional group (Table 3). Allograft was not used in the conventional group, though it was used in 14 cases (DBM putty (12 cases), one cortical span, and one spongiosa chip) in the Shark Screw^®^ group. Follow-up was 18 months in the conventional group and 10 months in the Shark Screw^®^ group.

### 3.2. Clinical Outcomes

Table 4 shows the outcome data. The union rate was 72.7% (8 of the 11 patients) in the conventional group and 96.7% (29 of the 30 patients) in the Shark Screw^®^ group. Time to union was significantly shorter in the Shark Screw^®^ group, with 11 weeks, in comparison to 39 weeks in the conventional group (*p* < 0.0001). Smoking delayed union in the conventional group, but it did not reach statistical significance, but not in the Shark Screw^®^ group (Supplementary Table S1). There was an earlier return to work for the Shark Screw^®^ group, with 12 weeks for the Shark Screw^®^ group and 25 weeks for the conventional group (*p* < 0.0001).

### 3.3. Complications

Complications were low in both groups, with four in the conventional group (two non-unions, one of them with osteonecrosis, one partial union, and one screw loosening) and one in the Shark Screw^®^ group (partial union). Non-unions were recorded in patients with ages of 35 and 59, whereas patients obtaining only partial unions were older (55 and 78 years of age). All patients with non-unions or only partial unions were non-smokers. Except for the 59-year-old patient (Lupus erythematosus) in the conventional group, none of the patients with complications had co-morbidities at the time of surgery. Metal removal in the conventional group was performed in seven of the eleven patients (64%) compared to one patient (3.4%) in the Shark Screw^®^ group. The one patient in the Shark Screw^®^ group had metal hardware from a previous surgery, which was left in place during revision surgery, but removed later.

### 3.4. Selected Case Descriptions

**Figure 2:**
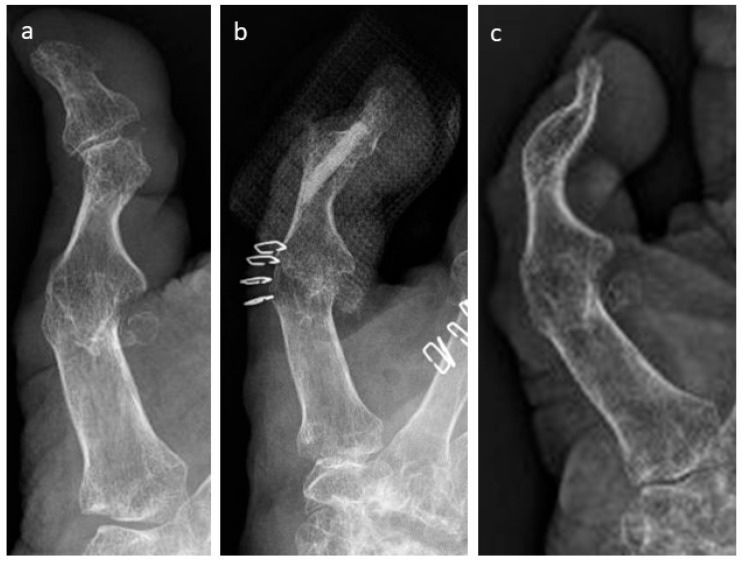
A failed thumb IP-arthrodesis of a patient with chronic polyarthritis is depicted. (**a**): presurgical X-ray; (**b**): the patient was treated with one Shark Screw^®^, and union was recorded after 12 weeks; (**c**): the Shark Screw^®^ was totally remodelled into the host bone after 21 months.

A failed thumb IP-arthrodesis of a patient with chronic polyarthritis is depicted in Figure 2a–c and was treated with one Shark Screw^®^; union was recorded after 12 weeks and the Shark Screw^®^ was totally remodelled into the host bone after 21 months (Figure 2c).

**Figure 3:**
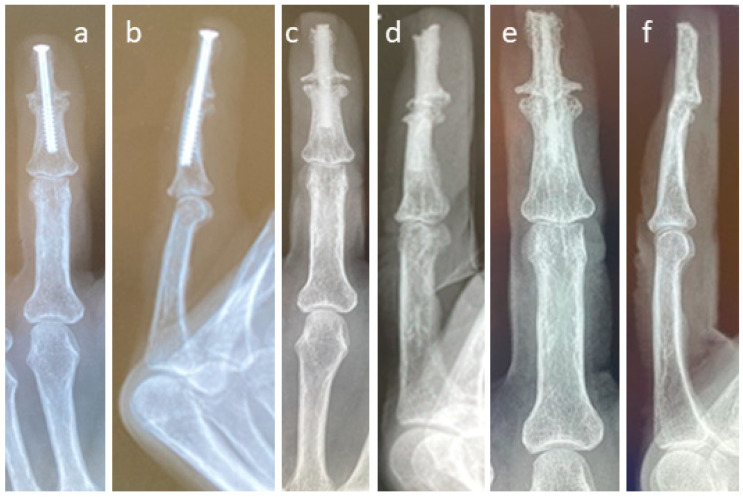
The revision after a failed arthrodesis of the middle finger with a metal screw. (**a**,**b**): The X-ray after the first treatment. Even hardware removal did not lead to union 31 months after surgery. (**c**,**d**): For the revision, one Shark Screw^®^ was placed, and union was obtained after 26 weeks. (**e**,**f**): 36 months after surgery, the Shark Screw^®^ is nearly totally remodelled.

The revision carried out on the left hand of a patient after a failed arthrodesis of a middle finger with a metal screw is shown in Figure 3. The X-ray after the first treatment is presented in Figure 3a,b. Even hardware removal did not lead to union 31 months after surgery. For the revision, one Shark Screw^®^ was placed (Figure 3c,d), and union was obtained after 26 weeks. 36 months after revision surgery, the Shark Screw^®^ is remodelled (Figure 3e,f).

**Figure 4:**
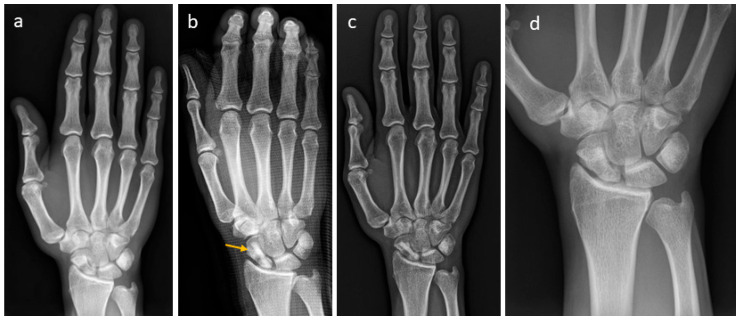
A patient with a non-union of the scaphoid. (**a**): presurgical X-ray; (**b**): the patient was treated with one Shark Screw^®^ (orange arrow); (**c**): union was obtained after eight weeks. (**d**): After 35 months, the Shark Screw^®^ is totally remodelled into host bone.

A patient with a non-union of the scaphoid is presented in Figure 4. Revision was performed with one Shark Screw^®^ (Figure 4b, orange arrow). Union was obtained after eight weeks (Figure 4c). After 35 months, the Shark Screw^®^ is totally remodelled into the host bone (Figure 4d).

**Figure 5:**
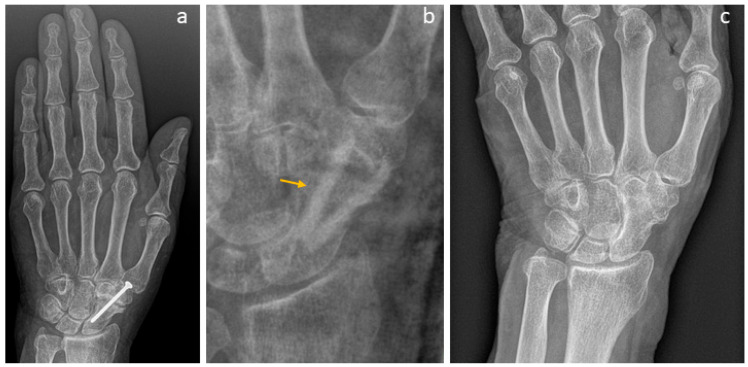
A patient was treated to overcome the non-union of the scaphoid–trapezoid–trapezium arthrodesis. (**a**): pre-revision X-ray; (**b**): the metal screw was removed, and the patient was treated with two Shark Screws^®^ (orange arrow)—union was detected eight weeks after revision surgery. (**c**): 14 months after revision, the Shark Screw^®^ is no longer visible.

To overcome the non-union (Figure 5a) of the scaphoid–trapezoid–trapezium arthrodesis, a patient was treated with two Shark Screws^®^ (Figure 5b, orange arrow). Union was detected eight weeks after revision surgery. A total of 14 months after revision, the Shark Screw^®^ is no longer visible (Figure 5c).

**Figure 6:**
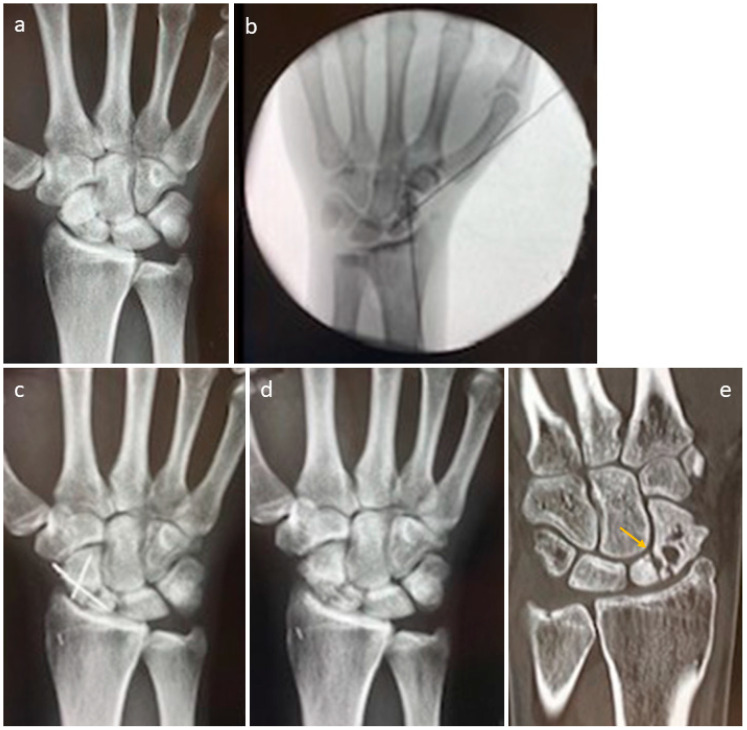
Non-union of the scaphoid after conventional treatment, even though autograft was used. (**a**): Pre-revision X-ray. (**b**): Intraoperative fluoroscopy using a vascularized femur condylar span which was attached to the aorta radialis. The span was stabilized with four K-wires. (**c**): Twelve weeks after surgery with the non-union still visible. (**d**): Shock-wave therapy did not improve the situation after eight months. (**e**): Five years after revision; the necrosis (orange arrow) of the proximal pole is visible, but the patient is nearly pain-free.

In Figure 6, we present the non-union obtained for the scaphoid after conventional treatment even though autograft was used. Figure 6a shows the pre-revision X-ray. Figure 6b depicts the intraoperative fluoroscopy using a vascularized femur condylar span which was attached to the aorta radialis. The span was stabilized with four K-wires. An X-ray taken 12 weeks after surgery is presented in Figure 6c, with the non-union still visible. Shock-wave therapy did not improve the situation after eight months (Figure 6d). Five years after revision, the necrosis of the proximal pole is visible (Figure 6e, orange arrow), but the patient is nearly pain-free (Figure 6e).

**Figure 7:**
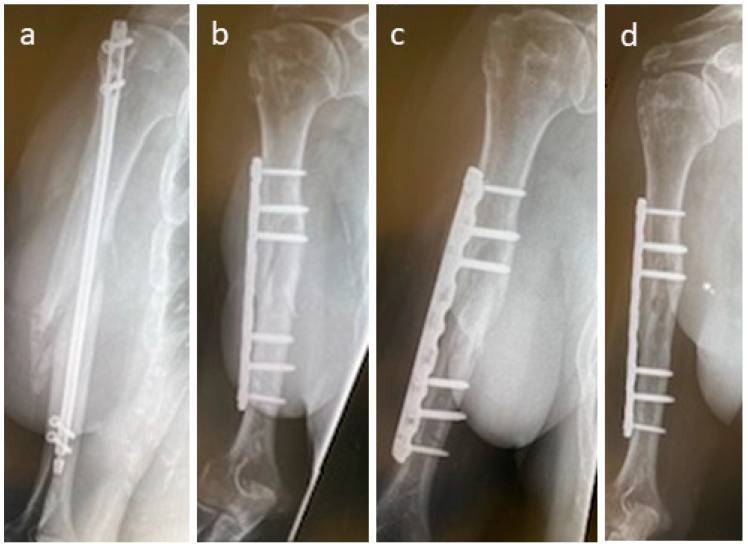
(**a**): A patient after non-union treatment after a fall at home. The first surgery was performed with a humerus nail T2 (Stryker), and 10 months after this surgery, no union was obtained. Revision was performed with a nine-hole plate and one 4 mm and one 4.5 mm Shark Screw^®^. (**b**): Four weeks after revision. (**c**): Eight weeks after revision, remodelling is visible. Union recorded 12 weeks post-revision. (**d**): One year post-revision, the Shark Screws^®^ are totally remodelled.

A patient was treated for non-union after surgery using a humerus nail after a fall at home (Figure 7); 10 months after initial surgery, non-union was recorded (Figure 7a). For revision, non-union debridement was performed, and the nail was removed and replaced with a nine-hole plate. Non-union was additionally bridged with two Shark Screws^®^. Four weeks after revision, the Shark Screws^®^ are still easily visible, and union is not yet observed (Figure 7b). Eight weeks after revision (Figure 7c), remodelling is visible in part of the former non-union area. Union was recorded 12 weeks after revision surgery. Figure 7d shows union and full remodelling of the Shark Screws^®^ one year post-revision.

**Figure 8:**
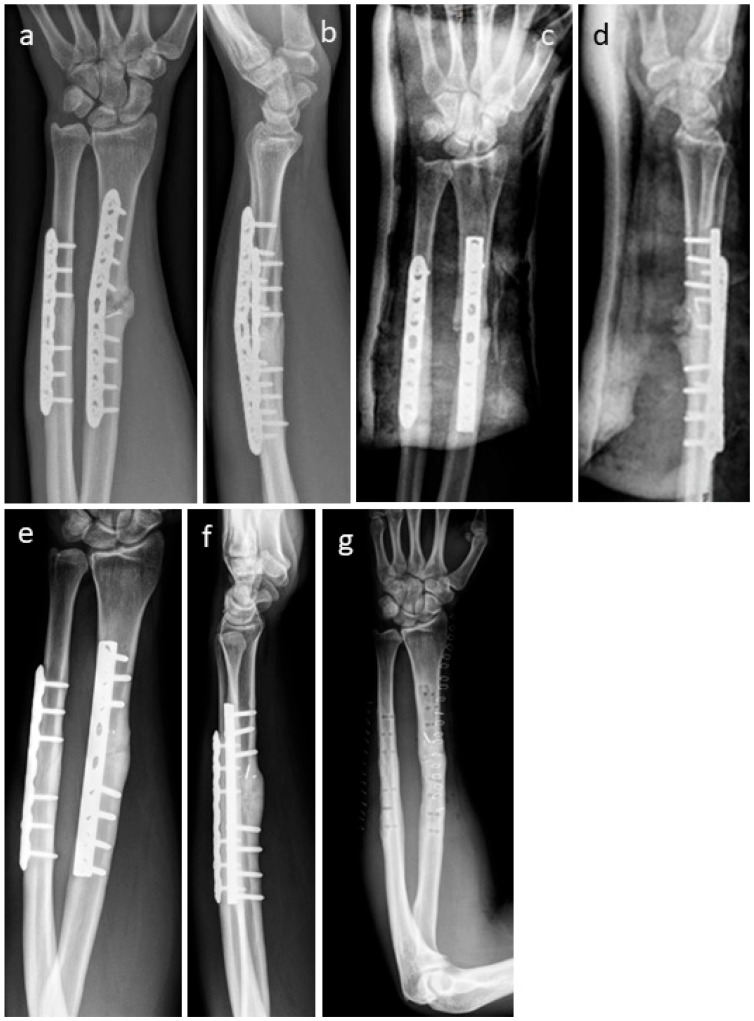
The non-union of a radius in a patient is shown; (**a**,**b**): pre-revision X-rays. As the former radius plate was bent, the radius was shortened in relation to the ulna. During the revision operation, the plate on the radius was replaced and the radius was restored to its original length. The resulting bone defect was filled with an allogeneic cortical block and DBM putty. In addition, the bone defect was stabilized and bridged with two Shark Screws^®^. (**c**,**d**): Post-surgery X-rays; union was obtained after 12 weeks. (**e**,**f**): Before metal hardware removal, one year after revision surgery. (**g**): After metal removal.

Figure 8 shows the non-union of a radius in a patient. As the former radius plate was bent, the radius was shortened in relation to the ulna (Figure 8a,b). During the revision operation, the plate on the radius was replaced and the radius was restored to its original length. The resulting bone defect was filled with an allogeneic cortical block and DBM putty. In addition, the bone defect was stabilized and bridged with two Shark Screws^®^ (Figure 8c,d). Union was obtained after 12 weeks; metal hardware was removed one year after revision surgery (Figure 8e,f), and an X-ray was taken after metal removal (Figure 8g).

**Figure 9:**
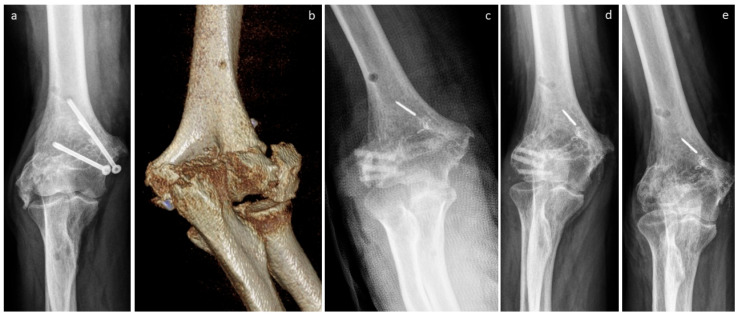
The non-union of the epicondyle of the lateral humerus of a patient. (**a**): Presurgical X-ray; (**b**): pre-surgical CT; (**c**): non-union was treated with three Shark Screws^®^. Union was observed after 10 weeks. (**d**): Four months after surgery. (**e**): A total of 21 months after revision, the Shark Screws^®^ are totally remodelled into the host bone.

The non-union of the epicondyle of the lateral humerus of a patient is presented in Figure 9. A pre-revision X-ray and CT scan are shown in Figure 9a,b. Non-union was treated with three Shark Screws^®^ (Figure 9c) and union was observed after 10 weeks. Figure 9d shows an X-ray taken after four months, with the Shark Screws^®^ totally remodelled into the host bone 21 months after revision (Figure 9e).

**Figure 10:**
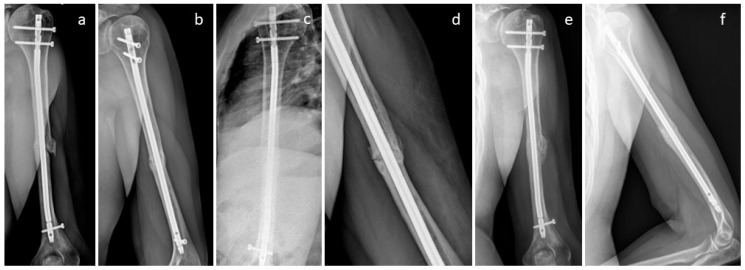
A non-union in the humerus shaft of a patient. (**a**,**b**): Presurgical X-rays. (**c**,**d**): The nail was left in, but the non-union was bridged with three Shark Screws^®^, as shown in the post-surgical X-rays; union was recorded after seven weeks. (**e**,**f**): Eight months after revision, the Shark Screws^®^ were remodelled into the host bone and are no longer visible.

A non-union in the humerus shaft of a patient is depicted in Figure 10b. The nail was left in, but the non-union was bridged with three Shark Screws^®^ (Figure 10c,d). Union was recorded after seven weeks. Eight months after revision, the Shark Screws^®^ were remodelled into the host bone and are no longer visible (Figure 10e,f).

**Figure 11:**
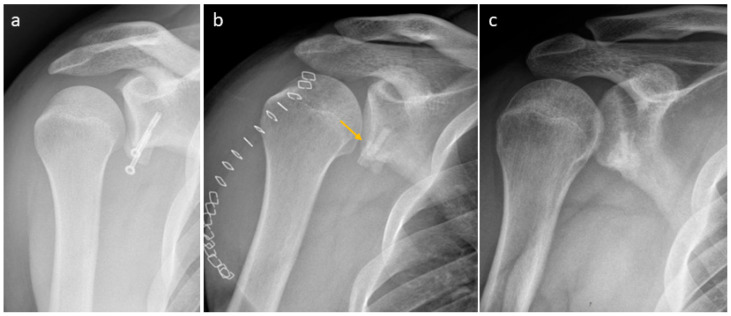
A failed Latarjet in a patient. (**a**): Pre-surgical X-ray; (**b**): the metal screws were taken out and the bone bed was prepared for the Shark Screw^®^ (drilling and thread cutting). One Shark Screw^®^ (orange arrow) was used for revision. Union was observed two months after surgery. (**c**): An X-ray taken four months after surgery shows that the Shark Screw^®^ is nearly remodelled into the host bone.

A failed Laterjet in a patient was treated with one Shark Screw^®^ (Figure 11). The metal screws were taken out (Figure 11a). No debridement of the non-union was performed, instead only preparing the bone bed for the Shark Screw^®^ (drilling and thread cutting). One Shark Screw^®^ (orange arrow) was used for revision (Figure 11b). Union was observed two months after surgery. Four months after surgery, the Shark Screw^®^ was totally remodelled into the host bone (Figure 11c).

**Figure 12:**
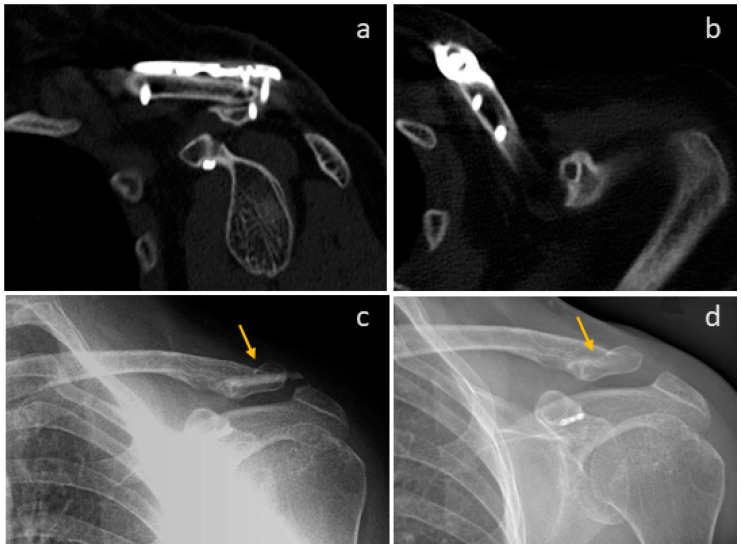
The revision of the non-union of a clavicula of a patient. (**a**,**b**): CT-scans: The former metal hardware was removed in the same session. (**c**): Two Shark Screws^®^ (orange arrows) were used for revision. 12 weeks after revision, bone union was recorded. (**d**): After 15 months, the Shark Screws^®^ are totally remodelled into the host bone.

In Figure 12, we show the revision of the non-union of a clavicula of a patient. Former metal hardware (Figure 12a,b) was removed in the same session, and two Shark Screws^®^ (orange arrow) were used for revision. Union was obtained 12 weeks after revision (Figure 12c). Fifteen months after revision, bone union is visible and the Shark Screws^®^ are totally remodelled into the host bone.

**Figure 13:**
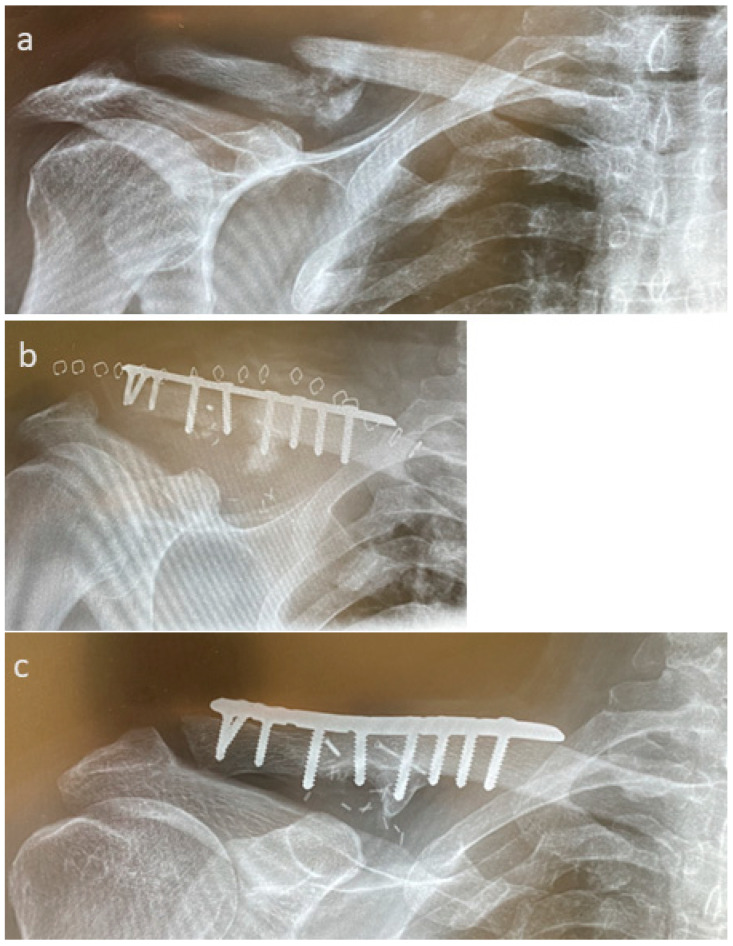
A case of non-union after a patient faced osteomyelitis after a fall at home. (**a**): Presurgical X-ray. (**b**): The fracture was anatomically reduced after pseudoarthrosis removal, the insertion of a microvascular pedicled MFC span, and bridging osteosynthesis with a 10-hole lateral clavicle plate (Arthrex, Naples, FL, USA). (**c**): Complete union one year after revision.

In Figure 13, we present the case of non-union after a patient faced osteomyelitis after a fall at home (Figure 13a). The fracture was anatomically reduced after pseudoarthrosis removal, inserting microvascular pedicled MFC spans and bridging osteosynthesis with a 10-hole lateral clavicle plate (Arthrex, Naples, FL, USA, Figure 13b), with complete union observed one year after revision (Figure 13c).

**Figure 14:**
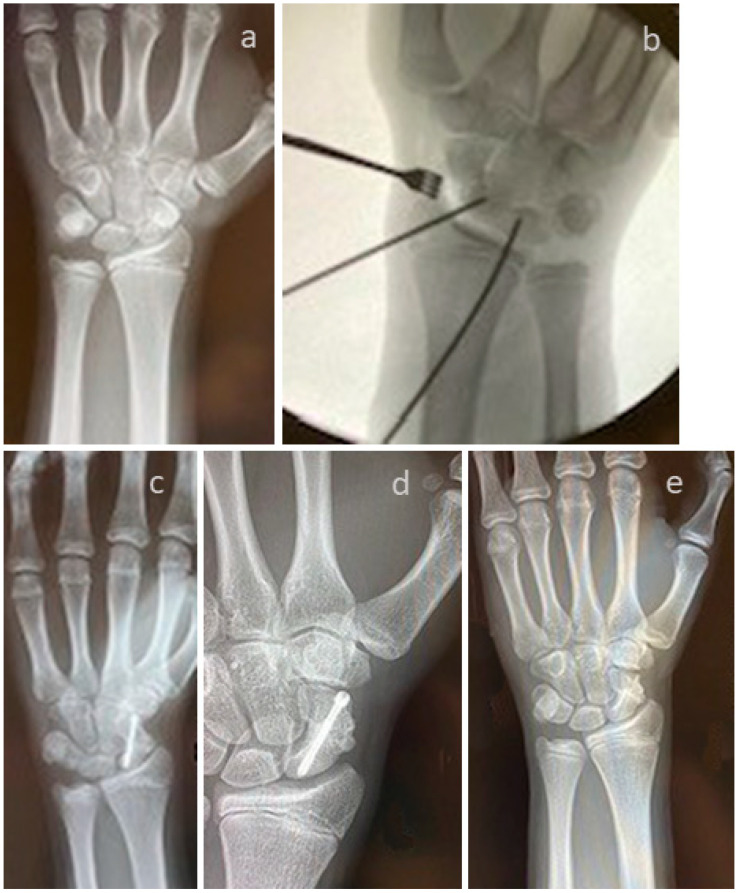
The case of a non-union of a scaphoid fracture with conservative treatment. (**a**): Pre-surgical X-ray; (**b**): interoperative fluoroscopy. Revision was performed using the Linscheid maneuver, with correction of the Humpback deformity, using an autologous iliac crest cortical bone span and a Herbert Screw. (**c**): X-ray two months after revision; (**d**): union was observed six months after surgery. Metal hardware was removed one year later. (**e**): X-ray 18 months post revision.

A case of a non-union of a scaphoid fracture with conservative treatment is shown in Figure 14. Revision was performed using the Linscheid maneuver, with correction of the Humpback deformity, using an autologous iliac crest cortical bone span and a Herbert Screw (Figure 14b). Figure 14c shows an X-ray taken after two months. Union was observed six months after surgery (Figure 14d). Metal hardware was removed one year later. Figure 14e shows an X-ray taken 18 months post-revision.

**Figure 15:**
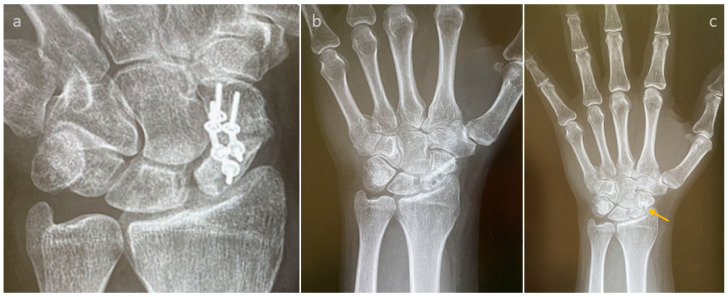
(**a**–**c**): A case of a patient with partial union after revision surgery in the Shark Screw^®^ group. Pseudoarthrosis after scaphoid fracture in the middle third after initial surgical treatment with angle-stable Medartis scaphoid plate (1.5 mm). (**a**): Revision was performed, including metal removal, elimination of the Humpback deformity, and pseudoarthrosis bridging with one 3.5 mm Shark Screw^®^. (**b**): six weeks after revision surgery. (**c**): Two years after revision, we recorded increasing callus formation, with the pseudoarthrosis still visible centrally, the Shark Screw^®^ (orange arrow) now completely resorbed, and the fracture not yet completely united.

A case of pseudoarthrosis after scaphoid fracture in the middle third after surgical treatment with an angle-stable Medartis scaphoid plate (1.5 mm) is presented in Figure 15. Revision was performed, including metal removal (Figure 15a), elimination of the Humpback deformity, and pseudoarthrosis bridging with one 3.5 mm Shark Screw^®^. Figure 15b represents the case six weeks after revision surgery. Two years after revision, we recorded increasing callus formation, with the pseudoarthrosis still visible centrally, the Shark Screw^®^ now completely resorbed, and the fracture not yet completely united (Figure 15c, orange arrow).

**Figure 16:**
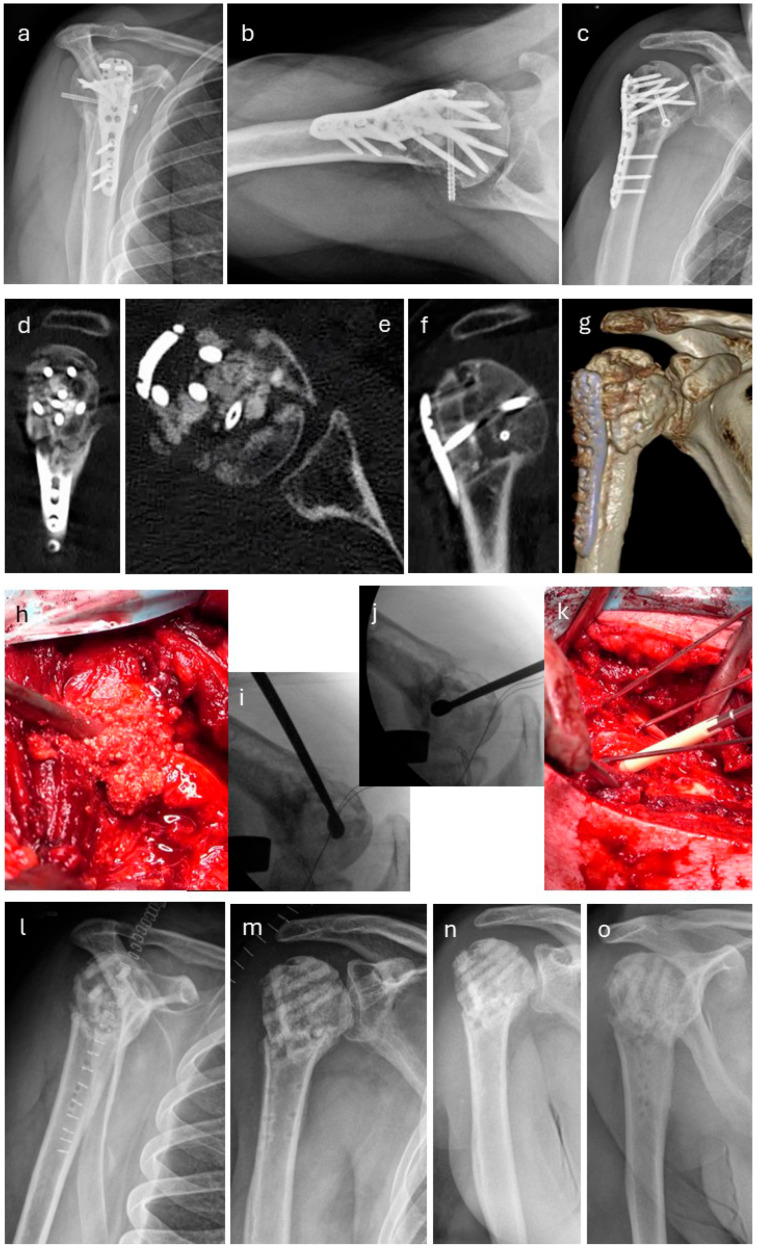
A case of a 44-year-old patient who had undergone surgery for a comminuted fracture of the right humerus, treated with a PHILOS^TM^ plate. (**a**–**c**): X-rays; (**a**): outlet view; (**b**): axial view; (**c**): anteroposterior view; the non-union failed to consolidate. (**d**–**f**): CT scans prior to revision; (**d**): sagittal view; (**e**): axial view; (**f**): coronal view; (**g**): CT 3D reconstruction; (**h**–**k**): eighteen months later, a single-stage procedure was performed, including removal of the bone substitute material (**h**), calcium phosphate, which, 18 months postoperatively, was non-vital, non-functional, and was not integrated into the patient’s bone metabolism, and hardware removal. (**i**,**j**): X-ray images during surgery demonstrate the extent of the defect cavity in the humeral head, depicted using a curette. (**k**): The pseudarthrosis is stabilized with five Kirschner wires, which are successively replaced by the transplantation of five human allogeneic cortical bone screws (Shark Screw^®^ DIVER) as cortical bone grafts for the bridging and fixation of the non-union, as well as the transplantation of human allogeneic bone granulate into the large humeral head defect. Follow-up radiographs (directly after surgery (**l**,**m**) and four weeks post-surgery (**n**,**o**)) after a fall onto the operated right shoulder show that the solely human biological osteosynthesis remains stable. The transplanted bone screws and the allogenic bone granulate have been partially remodelled by the host bone.

A 44-year-old patient who had undergone surgery for a comminuted fracture of the right humerus was treated with a PHILOS TM plate (Johnson&Johnson, Warsaw, IN, USA). Six months later, partial hardware removal and revision surgery were performed due to delayed bone healing. The non-union failed to consolidate. Eighteen months later, revision of the pseudarthrosis was performed. Figure 16a–g show a preoperative X-ray and 3D reconstruction before revision surgery. Figure 16h depicts the calcium phosphate particles found intraoperatively, which were present without reaction in the defect cavity; these were removed, and a single-stage procedure was performed (Figure 16h–k), including hardware removal. The pseudarthrosis was stabilized with five Kirschner wires (Figure 16k), which were successively replaced by five human allogeneic cortical bone screws (Shark Screw^®^ DIVER) as cortical bone grafts for the bridging and fixation of the non-union, as well as transplanting human allogeneic bone granulate into the large humeral head defect. The intraoperative X-rays (Figure 16i,j) visualize the extent of the humeral head defect, which is highlighted using a sharp curette. Follow-up radiographs directly after surgery (Figure 16l,m) and four weeks post-surgery (Figure 16n,o) after a fall onto the operated right shoulder show that the solely human biological osteosynthesis remains stable. The transplanted bone screws and the allogenic bone granulate have been partially remodelled by the host bone.

## 4. Discussion

The most important finding of this study is that the surgical treatment of non-union with the human allogeneic cortical bone screw (Shark Screw^®^) outperformed conventional treatment (metal hardware) with a 96.7% bone-healing rate, a shorter time to union (28 weeks earlier), and a faster return to work (14 weeks earlier).

As Elliott et al. [41] describes, the tissue that forms in and around a fracture should be considered a specific functional entity [41]. This ‘bone-healing unit’ produces a physiological response to its biological and mechanical environment, which leads to normal bone healing [41], which was confirmed for the human allogeneic cortical bone screw [33].

The Shark Screw^®^ group had fewer complications, with only one case of partial union recorded, which occurred after scaphoid revision surgery. An advantage of the Shark Screw^®^ is its avoidance of metal components, which may further cause problems. In two cases, the metal hardware from previous surgeries was left in; in one case, the old metal hardware was replaced with new metal hardware, and in the other, an ulna head prothesis was used in addition to the Shark Screws^®^ used. It is possible to combine the human allogeneic cortical bone screw with metal hardware. The use of the Shark Screw^®^ avoids donor-site morbidity and improves bone healing. The follow-up was significantly shorter in the Shark Screw^®^ group. This is because patients in whom union was confirmed did not have to come back for an additional visit. Because this is a retrospective study, patients were not contacted again after their last visit.

The non-union rate depends on the location of the fracture and is reported to be the highest in the shoulder, followed by the upper arm and the forearm, and is lowest in the hand [30]. In contrast, Reeh et al. describes that the hand has the highest probability for non-union [9]. The non-union rate in the humerus is reported to be between 7.2% [20] and 9% [31,42], and with a rate of 16% for non-union treatment in the humerus [4], 7% in the ulna [42], and 5% [42] in the radius. We recorded cases of non-union in the ulna and in the scaphoid, and partial union again in the ulna and in the scaphoid, which confirms the literature. Union rates for the revision of scaphoid non-union were reported to be between 50% and 100% [12,21,25,27,39,43,44,45]. Millrose et al. [29] describes a non-union rate between 3.6% and 8.6% for PIP joint arthrodesis depending on the technique used. The union rates in our cohort were 73% and 96.7% in the conventional group and in the Shark Screw^®^ group, respectively. In the conventional group, we had one non-union and one partial union in the ulna. Additionally, in the conventional group, there was one non-union in the pole fraction of the scaphoid, which is known to show higher non-union rates than for waist or distal fractures of the scaphoid [39,45]. In the Shark Screw^®^ group, we did not observe any non-union. Partial union in the Shark Screw^®^ group was recorded in one case of scaphoid (waist fraction) non-union revision.

Time to union for the clavicula after non-union was reported to be 3–8 weeks [46]. Literature reports treating forearm non-unions with allograft indicated that the treatments resulted in union after 102 days (14 weeks) and after 117 days (17 weeks) for the autograft group, when using plates and nails [13]. For the forearm, six months was reported for the time to union [11]. The time to union was 3.8 months without bone grafting [22]. After non-union revision, the time to union was noted after 3–4 months [25,39]. Others report 15 months until union was obtained for scaphoid fractures [45]. We could observe a significantly shorter time to union in the Shark Screw^®^ group (10.8 weeks), which was on the shorter end of the results described in the literature. Time to union in the conventional group was longer (39 weeks), with two patients (both smokers) displaying extremely long time to union (74 and 78 weeks). However, this value is still within the range described in the literature [45].

The complication rate using the conventional methods is reported to be between 4 and 33% for major complications [12,47] and up to 44% when including minor complications [47]. Lari et al. [48] reported in their review article a complication rate of 14% after distal radius fractures involving the volar rim. In our study, we recorded four complications (36%) in the conventional group, two non-unions, one partial union, and one case of screw loosening. In the Shark Screw^®^ group, there was only one complication (3.4%) of a partial union, which confirms the reports in the literature [49].

The hardware removal rates were reported to be 7.5% [50], 20% [12], 22% [48], and 65% [17]. Removal of internal fixation was noted to have an incidence of 1.92/100,000 person years and 20,385 cases between 2007 and 2019 [9]. In our patient cohort, the hardware removal rate was 63.6% in the conventional group and 3.4% (1 patient) in the Shark Screw^®^ group, which was significantly different (*p* < 0.0001). One patient in the Shark Screw^®^ group had hardware from previous surgeries, which was left in place during revision.

Rolo et al. [13] reported a return-to-work time of 9.4 months in the allograft group and 12.6 months in the autograft group when using metal plates in treating forearm non-unions [13]. The results described are longer than those observed in our study. After 4-corner fusion using bioabsorbable plates, return-to-work time was described to be 4.5 months [51], which is longer than our values in the Shark Screw^®^ group but shorter than those in our conventional group. After the fixation of scaphoid fractures with staples, return to work was reported to be 10 weeks [50], which is similar to our observation in the Shark Screw^®^ group, but primary fractures and non-unions were not separately analyzed. In our cohort, return to work was 14 weeks earlier in the Shark Screw^®^ group (11 weeks) than in the conventional group (25 weeks), which was statistically significant (*p* < 0.0001). Further studies should be conducted to support these findings.

Non-union is described more in male patients aged between 30 and 50 [9,42], which may be attributed to more high-energy trauma [6]. This was not true in our cohort. There were two males and two females in the group of partial unions and non-union. Reeh et al. [9] report that male patients under 30 years old are at the highest risk for non-union, whereas for females the risk of non-union increases with age. The latter statement was true for our patient cohort, because the two oldest patients with non-union/partial union were female, and the male patients with non-union/partial union were younger (35 and 55 years). We can confirm the accuracy of middle-age group having higher rates of non-unions in males (35 years) and the higher age in females (59 years), whereas the patients with partial unions were older (55 and 78 years). Ekegren et al. [31] state that patients between the age of 55–64 years are more than twice as likely to be resubmitted to the hospital for non-union than younger patients, which confirms our findings.

An interesting observation in our study is that 32% of the patients who were treated for non-union were smokers, whereas in the general European public, around 20% are smokers, with 28% in Turkey and 11% in the United States. Especially in the conventional group, the percentage of smokers was high (45%). We could not detect that time to union was significantly delayed for smokers [52].

The incidence of non-union in the upper arm and forearm is described to be 1.3/100,000 [9,53], and with 2.9–3.08/100,000 for the hand [9,53]. This adds up to 5824–13,798 patients per year in Europe. Walter et al. [53] describe a re-imbursement for non-union revision totaling between EUR 2900 (hand) and EUR 5095 (forearm). We observed a reduction in the non-union rate by 24% (*p* = 0.03853). We used a reduction of only 10% for our calculations. When using the human allogeneic cortical bone screw, the number of non-unions could be reduced by at least 10% (700 patients). Assuming EUR 4000 per reimbursement, this accumulates to EUR 2,800,000 per year for the health system in Europe. Additionally, this would mean savings for the health system, because there is no need for hardware removal (as described by Reeh et al. [9]), and savings for the patient with regard to lost wages.

### Limitation of the Study

The retrospective design of the study is a limitation. Fortunately, non-union occurs rarely, and thus the number of patients who agree to undergo revision is low. The involvement of multiple surgeons led to variations in surgical protocols; therefore, they are not described in detail in this study. Due to the positive impression obtained after the first few treatments with the human allogeneic cortical bone screw, the treatment with the conventional methods was employed less and less, and thus each group does not have a similar number of patients. Additionally, the patients in the conventional group are significantly younger, which should be kept in mind when interpreting the data.

## 5. Conclusions

In conclusion, the presented data show that the human allogeneic cortical bone screw alone or in combination with (remaining) metal hardware can be used for the non-union revision of a diversity of cases and results in shorter time to union and earlier return to work compared to the conventional treatment. The Shark Screw^®^ presents a reliable option for treating non-unions in the shoulder, forearm, hand, and fingers. The human allogeneic cortical bone screw combines both stability and biology within a single transplant. Further studies should be performed to underline these findings.

## Figures and Tables

**Table 1:** Demographic data.

	Conventional Treatment (Metal Hardware ± Graft)		Shark Screw^®^ Treatment (Shark Screw^®^ ± Metal Plate)	
	Number of patients	Mean ± SD	Number of patients	Mean ± SD
Number of patients	11	-	30	-
Age [years]	11	40.8 ± 19.9	30	56.5 ± 13.8 *
BMI [kg/m^2^]	11	25.1 ± 6.4	30	24.8 ± 5.2
Gender [male/female]	6/5	-	14/16	-
Smoker [yes/no]	5/6	-	7/23	-
Comorbidities [yes/no]	4/7	-	15/15	-
Non-union revision following:				
Surgical fracture treatment	3	-	12	-
Elective surgery (arthrodesis, osteotomy)	1	-	10	-
Conservative fracture treatment	7	-	8	-
Aseptic/septic	10/1	-	29/1	-

* *p* = 0.00669 *vs*. conventional group.

**Table 2:** Anatomic localization of non-union.

Anatomic Localization of Non-Union		Conventional Treatment (Metal Hardware ± Graft)	Shark Screw^®^ Treatment (Shark Screw^®^ ± Metal Plate)
	Number of patients	11	30
Shoulder	Clavicular	3	1
	Shoulder (Latarjet)	0	2
Arm	Humerus	1	4
	Ulna	1	2
	Radius	1	1
	Elbow	0	1
Hand	Scaphoid–Trapezoid–Trapezoidal (STT)	0	1
	Wrist	0	3
	Repetitive Strain Injury (RSI)	1	0
	Scaphoid	3	4
Finger	DIP1	0	2
	DIP2,3,4	0	1
	DIP2,3,5	0	1
	IP Thumb	0	4
	MCP 2	0	2
	MCP Thumb	0	1
	PIP3	1	0

**Table 3:** Clinical Data.

	Conventional Treatment (Metal Hardware ± Graft)	Shark Screw^®^ Treatment (Shark Screw^®^ ± Metal Plate)
	Number of patients	Number of patients
metal hardware use	11 (*per definition*)	-
additional to Shark Screw^®^	-	2 (1 × prothesis)
metal left in from previous surgery	-	2
Autograft use		
Iliac crest	2	-
Medial femoral condylar span (MFC)	2	-
Lateral femoral condylar span (LFC)	2	-
Allograft use		
DBM putty	0	12
cortical span	0	1
spongiosa chips	0	1

**Table 4:** Outcome data.

	Conventional Treatment (Metal Hardware ± Graft)		Shark Screw^®^ Treatment (Shark Screw^®^ ± Metal Plate)	
	Number of patients	Mean ± SD/%	Number of patients	Mean ± SD/%
Number of patients	11	-	30	-
Follow-up [weeks]	11	71.0 ± 41.0	30	42.6 ± 37.5 †
Complications [yes/no] [%]	4/7	36.4	1/29 *	3.4
Metal hardware removal [%]	7	63.6	1 ‡**	3.3
Bony union rate [%]	8	72.7	29	96.7
Partial union [%]	1	9.1	1	3.4
Non-union [%]	2	18.2	0	0.0
Time to union [weeks]	8	38.8 ± 26.3	29	10.8 ± 4.9 ***
Return to work [weeks]	10	25.3 ± 16.3	30	12.0 ± 5.5 ****

† *p* = 0.04226; * *p* = 0.00419; ** *p* < 0.0001; ‡ Hardware removal from previous surgery; *** *p* < 0.0001; **** *p* < 0.0001.

## Data Availability

Data are available in Supplementary Table S1.

## Supplementary Materials

The following supporting information can be downloaded at: https://www.mdpi.com/article/10.3390/life15091421/s1. Table S1: Upper extremity non-union treatment.

## Abbreviations

The following abbreviations are used in this manuscript:

DIP1	Distal interphalangeal joint finger 1
DIP2,3,4	Distal interphalangeal joint finger 2,3,4
DIP2,3,5	Distal interphalangeal joint finger 2,3,5
IP Thumb	Interphalangeal joint, thumb
MCP	Metacarpophalangeal joints
PIP3	Proximal interphalangeal joint finger 3
